# Associations between obesity (BMI and waist circumference) and socio-demographic factors, physical activity, dietary habits, life events, resilience, mood, perceived stress and hopelessness in healthy older Europeans

**DOI:** 10.1186/1471-2458-12-424

**Published:** 2012-06-11

**Authors:** Barbara Stewart-Knox, Maresa E Duffy, Brendan Bunting, Heather Parr, Maria Daniel Vas de Almeida, Mike Gibney

**Affiliations:** 1 Northern Ireland Centre for Food & Health (NICHE) School of Biomedical Sciences, University of Ulster, Coleraine, Northern Ireland; 2 Psychology Research Institute, University of Ulster, Coleraine, Northern Ireland; 3 University of Porto, Porto, Portugal; 4 University College Dublin, Dublin, Ireland

**Keywords:** Waist circumference, BMI, Obesity, Psychological well-being, Stress, Hopelessness, Resilience, Mood, Lifestyle, Life events, Survey, Lipgene

## Abstract

**Background:**

It is important to understand the psycho-social context of obesity to inform prevention and treatment of obesity at both the individual and public health level.

**Methods:**

Representative samples of middle-aged adults aged ≥43 years were recruited in Great Britain (GB) (n = 1182) and Portugal (n = 540) and interviewed to explore associations between body mass index (BMI), waist circumference (WC), demographic factors, physical activity, dietary habits (FFQ), life events (LES), Resilience (RS11), Mood (MS), Hopelessness (BDI) and Perceived Stress (PSS4). BMI (kg/m^2^) and WC (cm) were dependent variables in separate multiple linear regression models for which predictors were entered in 4 blocks: 1. demographic factors; 2. stressful life events; 3. diet/activity; and, 4. psychological measures.

**Results:**

In the GB sample, BMI (kg/m^2^) was predicted by less education, illness in a close friend or relative, frequent alcohol consumption and sedentary behaviour. Among the Portuguese, higher BMI (kg/m^2^) was predicted by lower resilience. Being male and less education were independent predictors of having a larger WC (cm) in both countries. Within GB, not working, illness in a close friend or relative, sedentary lifestyle and lower resilience were also independent predictors of a larger WC (cm).

**Conclusions:**

These data suggest that intervention to treat and/or prevent obesity should target males, particularly those who have left education early and seek to promote resilience. In GB, targeting those with high alcohol consumption and encouraging physical activity, particularly among those who have experienced illness in a close friend or relative may also be effective in reducing obesity.

## Background

Obesity rates and associated co-morbidity are increasing globally [1] and are attributed to detrimental lifestyle practices [2,3]. Socio-demographic factors appear to interplay with lifestyle to drive obesity. Obesity rates tend to be higher among the socioeconomically deprived [3-5] and the less educated [3,6-8]. There may be sex differences in how socio-demographic factors interact in obesity [4]. There is also some evidence to suggest that obesity rates are higher among those who have experienced adverse social circumstances [9]. There is growing interest in the psychology of health [10,11], lifestyle [12] and obesity [13]. That obesity is common among those diagnosed with clinical psychosis [14,15] has sparked the notion that obesity may be linked to psychological health and well-being. Previous studies of obesity and psychological well-being among healthy adults have almost exclusively considered depression and to a lesser extent stress. Evidence for an association between BMI (kg/m^2^) and depression [16-22] and/or stress [23,24] appears conflicting. Research which has considered waist circumference (WC) and depression and/or stress appears more consistent in that the majority of studies have indicated a link between greater WC (cm) and depression [17,18,25,26]. There are a few studies, however, which found no association between WC (cm) and depression [21-23]. What little research has considered WC (cm) and stress [23] has tended to produce null findings. These disparate findings imply that the relationship between obesity and psychological well-being is complex and worthy of further research.

Cultural differences in associations between well-being and obesity may go some way toward accounting for the conflicting findings reported in the literature. Whilst existing studies have taken place in a range of countries and cultures world-wide, there appears to be a lack of research which has compared more than one culture in the same study. There may also be sex differences in the way that wellbeing, particularly depression, interacts with obesity [16-18,26] and which may also explain the apparent disparity between studies. Previous studies of obesity and well-being have been biased toward the study of negative psychological factors to the neglect of positive traits that may be protective against obesity [11]. Wellbeing can be either *eudaimonic*, which is associated with one’s sense of autonomy, purpose and personal growth or *hedonic* which reflects life satisfaction [10,11]. Resilience, for example, is a positive trait which has been defined as ‘a dynamic process wherein individuals display positive adaptation despite experiences of significant adversity or trauma’ [27] and can be considered consistent with the eudaimonic model of wellbeing. Mood, on the other hand, can be construed as a central stable (hedonic) trait, which according to Underwood and Froming [28], comprises three dimensions, happiness/valance, strength/intensity and frequency/reactivity, all of which are associated with sociability. Our research, therefore, has considered not only depression and stress, but also resilience and trait mood. Previous research has also neglected to consider experiential factors that may impact upon well-being and obesity. Experience of stressful life events is likely to interact with resilience [29] and mood [28]. The aim of this study has been to determine, in two representative samples of older adults, whether there is a relationship between anthropometric factors (BMI and WC), lifestyle (frequency of food consumption and physical activity), demographic factors (sex and age), life experiences and psychological wellbeing (mood, resilience, stress and hopelessness) concurrently among healthy older people residing in Great-Britain (GB) and Portugal, whilst exploring interactions with sex. Given that previous studies have indicated that psychological factors interact differently with different anthropometric outcomes, separate models have been developed for BMI and WC anthropometric outcomes.

## Methods

The study was of a cross-sectional survey design conducted during autumn 2006.

### Sampling

Sample recruitment and data collection were undertaken by Ipsos Mori (GB), an international market research company. Nationally representative samples of adults over the age of forty-three years (N = 1722) were recruited in Great Britain (GB) (n = 1182) and Portugal (n = 540) (Table 1). Multi-stage stratified cluster sampling was undertaken using quotas in Great Britain (GB) for gender, social class, working status by gender and working status by region and in Portugal for age gender, location and town size. Each country was divided into regions or strata according to population size and density resulting in 210 sampling points in GB and 138 in Portugal. To ensure that the samples from each participating country were as nationally-representative as possible, responses were weighted by demographic factors for each sampling point based on the official statistics. The source for GB was the Office of National Statistics and for Portugal, INE National Institute of Statistics.

**Table 1:** Anthropometric characteristics between sex and country

	GB N = 1182		Portugal N = 540	
	Male	Female	Male	Female
	(n = 603)	(n = 579)	(n = 253)	(n = 287)
Height (M)	1.76 (0.07)	1.62 (0.07)	1.69 (0.07)	1.58 (0.06)
Waist Circum (cm)	93.6 (10.3)	84.3 (12.9)	98.85 (12.5)	93.6 (13.7)
BMI (kg /M²)	26.89 (4.39)	27.09 (4.79)	27.00 (3.61)	29.51 (5.12)

### The questionnaire

Review of the literature informed the selection of questionnaire items and scales. Items derived from validated scales were used under licence or with the permission of the authors. The resulting tool enquired as to demographic information; dietary habits; physical/sedentary activity; resilience; mood; hopelessness; perceived stress; and, life events. Given that a number of psychological variables were considered, to keep respondent fatigue at a minimum, validated short versions of scales were selected for inclusion in the questionnaire.

#### Food frequency questionnaire (FFQ)

A food frequency questionnaire (FFQ) was derived from that used previously in the Zenith study of healthy ageing [30] and then abridged, piloted and employed as a measure of how often ten different food groups (dairy; meat/poultry; fish/eggs; bread/cereals; confectionary/bakery; spreads; oils; fruit/vegetables; wine; and, other alcoholic drinks) were consumed. Responses were on a Likert scale ranging between 0–6 (never < 1 once per month; 1–3 per month; 1 per week; 2–6 per week; 1–3 times per day, 4 times per day, 5 or more per day). 'Exploratory factor analysis was conducted on the FFQ using Maximum Likelihood Extraction and Promax rotation. Three factors with eigenvalues >1, accounting for 47% of the total variance, indicated three distinct dietary profiles: Factor 1 - ‘Alcohol’ characterised by frequent alcohol (0.999) and wine (0.305) consumption; Factor 2 - ‘Unhealthy’ characterised by frequent consumption fat spreads (0.617), sweets/cakes/chocolate/biscuits (0.465), dairy produce (0.451) and bakery products (0.422); Factor 3 - ‘Healthy’ characterised by frequent intake of fish/eggs (0.459), vegetable oil (0.386) and wine (0.355) and infrequent intake of bakery products (−0.259). Based on this result each factor score (high alcohol; healthy; and, unhealthy) was entered into the regression analysis.

#### Physical activity

Physical activity and sedentary behaviour were assessed using a scale previously employed in the Zenith study [30]. Higher scores denoted greater sedentary behaviour on ‘weekdays’ and ‘weekends’ by indicating one of the following: On a week/weekend day (1) I spend up to 1 hour sitting down; (2) I spend over 1 to up to 4 hours sitting down; (3) I spend over 4 to up to 7 hours sitting down; (4) I spend over 7 to up to 9 hours sitting down; (5) I spend more than 9 hours sitting down; (6) None of these; (7) Don’t know/can’t remember. Physical activity was indicated in response to ‘within the last week I have spent at least 30 minutes on continuous physically activity (for example walking)’ (1) once or twice; (2) 3–5 times (3) 6–7 times (4) More than 7 times (5) None of these (6) Don’t know/ can’t remember. The equally weighted summed scores for weekend and weekday sedentary behaviour were entered into the regression analysis along with physical activity score.

#### Resilience scale (RS11)

The validated 11-item version of the 25-item Resilience Scale (RSS) developed by Wagnild and Young (1993) [31] was used to assess resilience. The full version (RSS) and the 11-item short (RS11) have been shown to have good internal consistency of between α 0.76 and 0.91 [31] and good concurrent validity [32]. Responses were on a 7-point Likert scale (agree/disagree) or ‘don’t know’. All 11 items loaded onto a single factor. Factor loadings for the items were as follows: ‘When I make plans I follow through with them’ (0.635); ‘I usually manage one way or another’ (0.69); ‘Keeping interested in things is important to me’ (0.744); ‘I am friends with myself’ (0.70); ‘I feel I can manage many things at a time’ (0.699); ‘I am determined’ (0.713); ‘I keep interested in things’ (0.755); ‘I can usually find something to laugh about’ (0.677); ‘I can usually look at a situation in a number of ways’ (0.697); ‘Sometimes I make myself do things whether I want to or not’ (0.488); ‘I have enough energy to do what I have to do’ (0.582). The model was adjusted using correlated errors (items 7 with 3; 7 with 6; 9 with 8; 10 with 4; 6 with 1, and, 2 with 1). The model fit statistics were as follows: CFI = 0.979; TLI = 0.969; RMSEA = 0.043; and, SRMR = 0.023. The reliability of the RS11 for the purpose of this study was α = .90. An equally weighted summed index was created from the 11 items and included within the regression analysis.

#### Mood survey

The validated 3-item version of the mood survey developed by Underwood and Froming (1980) [28] was employed to assess mood as a stable trait rather than as state. The items were: mood valance ‘How happy are you in general?’; mood variability ‘How frequently do your moods change?’; and, mood intensity ‘How intensely do you react to experiences?’. Responses were on a 9-point Likert scale or ‘don’t know’. Each item was entered individually into the regression analysis.

#### Beck hopelessness scale (BHS)

This 20-item Beck Hopelessness Scale (BHS) was originally developed by Beck and colleagues in 1974 [33] to measure the extent of negative expectations/attitudes relating to the immediate and long-term future. The BHS has been shown to have good internal consistency (α 0.82-0.93) and good predictive validity (0.91) [33]. On the basis of factor structure [33], eight items were derived from the BHS and included in the model. Responses were dichotomous (agree/disagree) or ‘don’t know’. A one factor confirmatory model was tested by weighted least squares means and variances (CFI = 0.948 and TLI = 0.965; RMSEA = 0.028): ‘I might as well give up because there is nothing I can do about making things better for myself’ (0.693); ‘When things are going badly, I am helped by knowing that they cannot stay that way forever’ (−0.322); ‘I just can’t get opportunities and there’s no reason I will in the future’ (0.809); ‘All I can see ahead of me is unpleasantness rather than pleasantness’ (0.716); ‘I don’t expect to get what I really want’ (0.627); ‘I never get what I want so it’s foolish to want anything’ (0.741); ‘It’s very unlikely that I will get any real satisfaction in the future’ (0.775); ‘There’s no use in really trying to get anything I want because I probably won’t get it’ (0.734). Scores from all 8 items were summed into an equally weighted index and included in the regression analysis.

#### Perceived stress scale (PSS)

The validated 4-item version [34] of the Perceived Stress Scale PSS scale devised by Cohen and colleagues (1983) [35] has been used for this study. Responses were on a 75-point Likert scale ‘never-very often’ or ‘don’t know’. A two factor model was used to describe the data. Two items loaded onto factor 1 ‘emotional stress’: ‘In the last month, how often have you felt confident about your ability to handle your personal problems?’ (0.870); ‘In the last month, how often have you felt that things were going your way?’ (0.636). Two items loaded onto factor 2 ‘stress control’: ‘In the last month, how often have you felt that you were unable to control the important things in your life?’ (0.719); ‘In the last month, how often have you felt difficulties were piling up so high that you could not overcome them?’ (0.782). The correlation coefficient between the two factors was 0.397. The overall reliability of the four items was α = 0.71. An equally weighted summed index of the items, which loaded onto each of the factors, was entered into the linear regression analysis as one variable.

#### Life events scale (LES)

The 10-item Life Events Scale (LES) developed by Mooy *et al*. (2000) [36] was used to record the occurrence (or not) of: (a) serious or long lasting problems with a partner; (b) end of a significant or long-term relationship; (c) serious or long-term financial problems; (d) personal long-term illness or disability; (e) personal long-term illness or disability lasting more than six months; (f) serious or long-term illness or disability of a child; (g) serious or long lasting social or behavioural problems with a child; (h) death of someone close to you (child/partner/sibling/close friend); (i) Forced job change/loss of job/retirement; and, (j) serious or long lasting stress at work. These dichotomous items were collapsed into five variables semantically described as: ‘relationship problems’ (a and b); ‘financial difficulties’ (c); ‘illnesses’ (d, e, f and g); ‘bereavement’ (h); and, ‘job difficulties’ (i and j) which were entered into the multiple regression analysis.

### Procedure

Ethical approval for the survey was granted by the Office of Research Ethics Committee Northern Ireland (ORECNI). Fieldworkers in both countries were trained and in the employment of the market research company (IPSOS Mori). Respondents provided written (informed) consent before taking part. Omnibus survey employing a representative sample recruited in Great Britain and Portugal was undertaken during January 2006. Data were collected in each individual’s own home by interview. Cue cards were used to present the response options for each item. Anthropometric measurements were taken following completion of the questionnaire. Waist circumference was measured by means of a tape measure at the level of the umbilicus in males and at the narrowest diameter in females. BMI (kg/m²) was calculated from height measured by stadiometer and body weight by an electronic scale.

### Statistical analysis

Statistical analyses were undertaken using the SPSS (Version 19.0; SPSS UK Ltd, Chertsey, United Kingdom). Two-way MANOVA was employed to look at differences in anthropometric and demographic characteristics, height (m), WC (cm), BMI (kg/m^2^) and age (years) between country and sex. Associations between the independent variables (Resilience Scale, Mood Survey, Beck Hopelessness Scale and Perceived Stress Scale (emotional stress and stress control scores) and the dependent variables WC (cm) and BMI (kg/m^2^) were examined by multiple linear regression analysis. The GB and Portugal samples were analysed separately to establish which independent variables were the best predictors of BMI (kg/m^2^) and WC (cm) within each country. The aim of the analysis was to establish the degree to which the psychological factors explained/contributed to the variance in BMI (kg/m^2^) and WC. Demographic information (age; sex; education level), life events (LES) and lifestyle factors (Dietary habits (FFQ) and physical activity (PAQ)) thus were entered first into the analysis so that each could be controlled prior to entry of the psychometric data. The predictors were entered simultaneously in 4 blocks (1. demographic factors; 2. life events; 3. diet/activity; 4. psychological measures). Continuous measures (age, years of education and psychometric scores) were adjusted to assess if there were any potential interactions with sex. Variables were centred by subtracting the overall means from each individual value and then multiplying by sex to establish if there were any interactions with sex. Mahalanobis distance values indicated that there were no multivariate outliers among the independent variables (i.e. no values greater that the chi-squared value of 37.65) [37]. The adjusted R² values, Beta values and standard error have been presented for each country (i.e. GB and Portugal).

## Results

### Sample description

The sample (N = 1722) comprised in GB (n = 1182) 51% (n = 603) male and 49% (n = 579) female and in Portugal (n = 540) 47% (n = 253) male and 53% (n = 287) female. The age range for the total sample was 43–93 years (Table 1). Mean years spent in education among the GB males was 10.77 (sd.8.38) and females was 9.05 (sd.8.38). Among the Portuguese males the mean years spent in education was 5.03 (sd.2.08) and for females it was 4.13 (sd.2.51). This relatively limited amount of time spent in education is typical among the older population in Portugal.

GB males had a smaller WC (cm) than Portuguese males (P = 0.0001) and GB females had a smaller WC (cm) than Portuguese females (P = 0.0001) (F = (3,1704) = 105.94, P = 0.0001). There were no differences in BMI (kg/m^2^) between respondents in Portugal and GB. There were no within sex differences in BMI (F(3,1713) = 1.32, P = 0.26) (Table 1).

### Anthropometric, demographic factors, lifestyle and wellbeing: GB sample

#### BMI (kg/m^2^)

Fewer years spent in education (β-0.12, P = 0.01) accounted for 1% of the variance in BMI (kg/m^2^) (F(6,926) = 2.36, P = 0.03). Having reported ‘illness-related’ life events (β = −0.14. P = 0.0001) explained an further 2% (i.e. 3%) of the variance in BMI (kg/m^2^) (F(11,921) = 3.36, P = 0.000). Having a high alcohol consumption (β = −0.07, P = 0.04) and sedentary behaviour (β = 0.14, P = 0.0001) contributed a further 2% (i.e. 5%) of the variance in BMI (kg/m^2^) (F(16, 916) = 3.91, P = 0.0001). The inclusion of the psychological variables within model 4 did not further explain the variance in BMI (kg/m^2^) but improved the model to 6% (F(26,906) = 3.07, P = 0.0001) (Table 2).

**Table 2:** **Multiple linear regression analysis predicting BMI (kg/m**^**2**^**) from demographic factors, life events, food habits, physical activity and psychological factors in the Great Britain sample (N = 1182)**

**Demographics**	**Model 1**			**Model 2**			**Model 3**			**Model 4**		
	**B**	**SE**	**Beta**	**B**	**SE**	**Beta**	**B**	**SE**	**Beta**	**B**	**SE**	**Beta**
Sex	−0.28	0.31	−0.03	−0.38	0.32	−0.04	−0.24	0.33	−0.03	−0.36	0.33	−0.38
Working Status	−0.17	0.38	−0.02	−0.48	0.39	−0.05	−0.63	0.38	−0.07	−0.56	0.38	−0.06
Age (years)	−0.00	0.02	−0.00	0.00	0.02	0.001	−0.01	0.03	−0.02	−0.01	0.02	−0.02
Ed (yrs)	−0.07	0.03	**−0.12**	−0.06	0.03	**−0.11**	−0.06	0.03	**−0.10**	−0.06	0.03	−0.10*
Age by*sex	−0.04	0.03	−0.06	−0.03	0.03	−0.06	−0.25	0.03	−0.04	−0.64	0.03	−0.06
Ed (yrs) by*sex	0.03	0.04	0.03	0.03	0.04	0.02	−0.02	0.04	0.03	0.03	0.04	0.04
**Life Events**												
Relationships				0.72	0.72	−0.04	−0.65	0.72	−0.04	−0.43	0.72	−0.03
Financial				0.93	0.93	−0.03	−0.78	0.93	−0.04	−0.87	0.93	−0.05
Illness				0.36	0.36	**0.14**	1.19	0.36	**0.11**	1.37	0.36	0.13**
Bereavement				0.35	0.35	0.006	0.09	0.35	0.01	0.15	0.35	0.01
Employment				0.52	0.53	0.04	0.54	0.51	0.04	0.58	0.52	0.04
**Lifestyle**												
SED activity 1 ^a^							−0.38	0.09	**0.12**	0.37	0.09	0.13**
PA activity 2 ^b^							−0.07	0.11	−0.02	−0.04	0.11	−0.01
FFQ (alcohol)							−0.33	0.16	**−0.07**	−0.34	0.16	**−0.07** †
FFQ (unhealthy)							0.11	0.19	0.02	0.08	0.19	0.02
FFQ (healthy)							−0.24	0.16	−0.05	−0.27	0.16	**−0.06** †
**Wellbeing**												
General Mood										0.24	0.16	0.08
Intense Mood										0.12	0.11	0.05
Variable Mood										−0.02	0.12	−0.01
Hopelessness										0.05	0.32	−0.05
Resilience										−0.03	0.03	−0.05
Emotional stress										−0.09	0.15	−0.03
Stress control										−0.11	0.13	−0.05
General mood **¹**										−0.12	0.22	−0.03
Intense Mood **¹**										0.17	0.96	0.07
Variable Mood **¹**										−0.04	0.16	−0.01
Hopelessness **¹**										0.04	0.44	0.00
Resilience **¹**										−0.03	0.04	−0.04
Emotional stress **¹**										−0.26	0.21	−0.06
Stress control **¹**										−0.09	0.19	−0.02
**Adjusted R²**		**0.01**			**0.03**			**0.05**			**0.06**	

*P < 0.05, **P < 0.01, †10% significant.

Abbreviations: *SED* Sedentary, *PA* Physical activity, *FFQ* Food Frequency Questionnaire.

**¹** = controlled for sex.

^a^ Sedentary behaviour at weekdays & weekends; ^b^ Physical activity in the previous week.

#### WC (cm)

Demographic factors (model 1) including, sex (being male) (β = 0.38, P = 0.0001), fewer years in education (β = −0.12, P = 0.009), age*sex (being older and male) (β = 0.11, P = 0.02) and working status (unemployed) (β = −0.08, P = 0.02) accounted for 16% of the variance in WC (F(6,910) =30.692, P = 0.0001). When life events (model 2) were added to the model, ‘illness-related’ events predicted a larger WC (cm) (β = 0.15, P = 0.0001), which together with demographic factors explained a further 2% of the variance (i.e. 18%) in WC (cm) (F(=11, 905) = 19.39, P = 0.0001). The incorporation of lifestyle factors (diet/physical activity) (model 3) indicated that greater sedentary behaviour predicted a larger WC (β = 0.14, P = 0.0001), contributing an extra 2% of the variance (i.e. 20%) in WC (F = (16, 900) = 15.17, P = 0.0001). The addition of psychological measures (model 4) to the regression model, indicated that lower scores on resilience (ß = −0.11, P = 0.04) predicted a larger WC (cm). Resilience, therefore, was the only significant psychological predictor variable in the final model (four) which explained 20% of the variance in WC (cm) (F(28, 916) = 9.18, P = 0.0001). Sex had no influence upon psychological variables in determining WC (cm) (Table 3).

**Table 3:** Multiple linear regression analysis predicting waist circumference (cm) (WC) from demographic factors, life events, food habits, physical activity and psychological factors in the Great Britain sample (N = 1182)

**Demographics**	**Model 1**			**Model 2**			**Model 3**			**Model 4**		
	**b**	**SE b**	**ß**	**b**	**SE b**	**ß**	**b**	**SE b**	**ß**	**b**	**SE b**	**ß**
Sex	9.58	0.77	0.38**	9.40	0.77	0.37**	9.48	0.81	0.37**	9.23	0.82	0.36**
Working Status	2.3	0.94	0.08**	1.38	0.95	0.05	1.05	0.94	0.04	1.21	0.94	0.05
Age (years)	−0.06	0.06	−0.05	−0.03	0.06	−0.03	−0.06	0.06	−0.05	−0.05	0.06	−0.05
Ed (yrs)	−0.19	0.07	−0.13**	−0.18	0.07	−0.12**	−0.16	0.07	−0.11*	−0.17	0.07	−0.11**
Age by*sex	0.19	0.07	0.12*	0.20	0.07	0.12*	0.22	0.07	0.14**	0.20	0.07	0.13**
Ed (yrs) by*sex	0.11	0.10	0.05	0.10	0.09	0.05	0.10	0.09	0.05	0.12	0.10	0.05
**Life Events**												
Relationships				−1.09	1.77	−0.03	−1.24	1.76	−0.03	−0.78	1.77	−0.02
Financial				1.03	2.28	0.02	0.63	2.27	0.01	0.52	2.28	0.01
Illness				4.34	0.88	0.15**	3.58	0.88	0.13**	3.95	0.90	0.14**
Bereavement				0.05	0.87	0.00	0.09	0.86	0.00	0.30	0.87	0.01
Employment				0.86	1.27	0.02	0.80	1.25	0.02	0.88	1.28	0.02
**Lifestyle**												
SED activity 1 ^a^							1.02	0.23	0.14**	0.97	0.24	0.13**
PA activity 2 ^b^							0.19	0.28	0.02	0.22	0.28	0.02
FFQ (alcohol)							−0.35	0.40	−0.03	−0.38	0.40	−0.03
FFQ (unhealthy)							0.01	0.45	0.00	−0.02	0.45	0.00
FFQ (healthy)							−0.30	0.39	−0.02	−0.39	0.39	−0.03
**Wellbeing**												
Happy Mood										0.68	0.38	**0.08†**
Intense Mood										0.35	0.27	**0.09†**
Variable Mood										−0.02	0.28	−0.00
Hopelessness										−0.06	0.78	0.00
Resilience										−0.15	0.06	−0.11*
Emotional stress										−0.11	0.37	−0.01
Stress control										−0.33	0.22	−0.07
General Mood **¹**										−0.35	0.55	−0.03
Intense Mood **¹**										−0.15	0.39	−0.02
Hopelessness **¹**										−0.01	1.07	0.00
Resilience **¹**										0.06	0.10	0.03
Emotional Stress **¹**										−0.40	0.52	−0.13
Stress control **¹**										0.14	0.45	0.02
**Adjusted R²**		**0.16**			**0.18**			**0.20**			**0.20**	

*P < 0.05, **P < 0.01, †10% significant.

Abbreviations: *SED* Sedentary, *PA* Physical activity, *FFQ* Food Frequency Questionnaire.

**¹** = controlled for sex.

^a^ Sedentary behaviour at weekdays & weekends; ^b^ Physical activity in the previous week.

### Anthropometric, Demographic Factors, Lifestyle and Wellbeing: Portuguese Sample

#### BMI (kg/m^2)^

Higher BMI (kg/m^2^) was associated with older age (ß = −0.224, P = 0.05) and lower resilience scores (ß = −0.25, P = 0.05) (model 4) accounting for 1% of the variance in BMI (Table 4). Demographic characteristics, lifestyle factors and life events did not contribute significantly (P = 0.05) to the model.

**Table 4:** **Multiple linear regression analysis predicting BMI (kg/m**^**2)**^**from demographic factors, life events, food habits, physical activity and psychological factors in a Portuguese sample (N = 540)**

**Demographics**	**Model 1**			**Model 2**			**Model 3**			**Model 4**		
	**B**	**SE**	**Beta**	**B**	**SE**	**Beta**	**B**	**SE**	**Beta**	**B**	**SE**	**Beta**
Sex	-0.55	0.57	-0.06	-0.67	0.58	-0.08	-0.90	0.66	-0.11	-0.78	0.70	-0.09
Working Status	0.01	0.63	0.00	-0.17	0.64	-0.02	-0.16	0.64	-.018	-0.10	0.65	-0.01
Age (yrs)	-0.05	0.04	-0.14	-0.06	0.04	-0.15	-0.07	0.04	**-0.19**†	-0.08	0.04	-0.22*
Ed (yrs)	-0.12	0.17	-0.07	-0.10	0.17	-0.05	-0.12	0.17	-0.06	-0.16	0.18	-0.08
Age by*sex	0.06	0.05	0.12	0.06	0.05	0.12	0.07	0.05	0.14	0.09	0.05	0.17
Ed (yrs) by*sex	0.24	0.25	0.08	0.25	0.26	0.09	0.29	0.26	0.10	0.29	0.28	0.10
**Life Events**												
Relationships				-0.49	2.02	-0.03	-0.42	2.03	-0.02	-0.60	2.11	-0.03
Financial				1.24	2.51	0.05	1.55	2.54	0.06	1.32	2.67	0.05
Illness				1.28	0.70	**0.12**†	1.10	0.70	0.11	1.10	0.72	**0.11** †
Bereavement				-0.65	0.76	-0.05	-0.56	0.76	-0.05	-0.62	0.80	-0.05
Employment				-0.98	1.23	-0.05	−1.05	1.23	-0.06	−1.01	1.27	-0.05
**Lifestyle**												
SED activity 1^a^							0.18	0.34	0.04	0.28	0.21	0.09
PA activity 2^b^							-0.07	0.32	**-0.02**†	-0.52	0.30	**-0.12** †
FFQ (alcohol)							-0.17	0.33	-0.03	0.29	0.36	0.06
FFQ (unhealthy)							0.30	0.20	0.10	-0.06	0.33	-0.01
FFQ (healthy)							-0.50	0.28	-0.11	-0.16	0.35	-0.03
**Wellbeing**												
General Mood										-0.19	0.31	-0.07
Variable Mood										0.03	0.21	0.01
Intense Mood										-0.03	0.25	-0.01
Hopelessness										0.06	0.38	0.02
Resilience										0.09	0.05	0.25*
Emotional Stress										0.36	0.19	**0.24 †**
Stress control										0.57	0. 40	0.04
General mood **¹**										0.21	0.44	0.05
Variable mood **¹**										0.58	2.97	0.19
Intense mood **¹**										0.24	0.34	0.07
Hopelessness **¹**										−0.81	0.61	−0.12
Resilience**¹**										−0.11	0.07	−**0.22†**
Emotional stress **¹**										−0.15	0.24	−0.04
Stress control **¹**										−0.57	0. 40	−0.17
**Adjusted R ²**		**0.01**			**0.01**			**0.00**			**0.01**	

*P < 0.05, **P < 0.01, †10% significant.

Abbreviations: *SED* Sedentary, *PA* Physical activity, *FFQ* Food Frequency Questionnaire.

**¹** = controlled for sex.

^a^ Sedentary behaviour at weekdays & weekends; ^b^ Physical activity in the previous week.

#### WC (cm)

Multiple regression analysis indicated that demographic factors (model 1) including, sex (being male) (ß = 0.21, P = 0.001) and fewer years of education (ß = −0.19, P = 0.03) accounted for 7% of the variance in WC (cm) (F(6,260) = 4.405, P = 0.0001). The incorporation of stressful life events (model 2) reduced the variance in WC (cm) by 1% (6%) (F(11, 255) = 2.54, P = 0.005). Lifestyle factors (model 3) contributed an extra 1% (7%) (F(16, 257) = 3.08, P = 0.005), indicating that greater physical activity predicted a smaller WC (ß = −0.12, P = 0.03). Finally, the incorporation of psychological variables (model 4) weakened the model and reduced variance in WC (cm) by 1% from model 3 (6%) (F(28,238) = 1.63, P = 0.03) (Table 5).

**Table 5:** Multiple linear regression analysis predicting waist circumference (cm) (WC) from demographic factors, life events, food habits, physical activity and psychological factors in a Portuguese sample (N = 540)

**Demographics**	**Model 1**			**Model 2**			**Model 3**			**Model 4**		
	**B**	**SE**	**Beta**	**B**	**SE**	**Beta**	**B**	**SE**	**Beta**	**B**	**SE**	**Beta**
Sex	5.54	1.67	0.21**	5.63	1.70	0.21**	4.49	1.93	0.17*	4.70	1.97	0.17*
Working Status	−0.53	1.95	−0.02	−0.56	1.98	−0.02	−0.54	1.97	−0.02	−0.61	1.99	−0.02
Age (yrs)	0.39	0.11	0.03	0.02	0.11	0.01	−0.03	0.12	−0.03	−0.04	0.12	−0.04
Ed (yrs)	−1.07	0.49	−0.19*	−1.04	0.50	−0.18*	−1.02	0.50	−0.18*	−0.83	0.52	−0.14
Age by*sex	0.26	0.15	**0.16†**	0.28	0.15	**0.17†**	0.28	0.15	**0.17†**	0.32	0.16	0.19*
Ed (yrs) by*sex	1.01	0.77	0.11	1.07	0.77	0.11	1.08	0.78	0.12	0.85	0.82	0.09
**Life Events**												
Relationships				−1.99	4.72	−0.03	−2.52	4.73	−0.04	−4.88	4.90	−0.08
Financial				2.14	7.05	0.02	3.78	7.08	0.04	6.13	7.21	0.07
Illness				2.22	2.11	0.07	1.87	2.11	0.06	1.60	2.18	0.05
Bereavement				0.87	2.24	0.02	0.69	2.25	0.02	0.94	2.34	0.03
Employment				−2.82	3.85	−0.04	−3.31	3.84	−0.05	−3.26	3.91	−0.05
**Lifestyle**												
SED activity 1^a^							0.82	0.53	0.10	0.63	0.54	0.08
PA activity 2^b^							−1.70	0.86	−0.12*	−1.62	0.90	**−0.12†**
FFQ (alcohol)							0.83	0.99	0.06	0.69	1.04	0.05
FFQ (unhealthy)							−0.51	0.93	−0.04	−0.82	0.96	−0.06
FFQ (healthy)							0.52	0.88	0.04	0.26	0.92	0.02
**Wellbeing**												
General Mood										−0.78	0.84	−0.09
Intense Mood										−0.09	0.69	−0.01
Variable Mood										1.03	0.61	**0.15†**
Hopelessness										−1.44	1.14	−0.11
Resilience										−0.02	0.13	−0.02
Emotional Stress										0.60	0.86	0.08
Stress control										0.82	0.82	0.11
General mood **¹**										1.03	1.28	0.43
Variable mood **¹**										−0.40	0.88	−0.04
Intense mood **¹**										−0.45	1.01	−0.04
Hopelessness **¹**										0.33	1.69	0.18
Resilience **¹**										0.08	0.18	0.05
Emotional stress **¹**										−0.15	1.20	−0.01
Stress control **¹**										−0.08	1.21	−0.01
**Adjusted R ²**		**0.07**			**0.06**			**0.07**			**0.06**	

*P < 0.05, **P < 0.01, †10% significant.

Abbreviations: *SED* Sedentary, *PA* Physical activity; *FFQ* Food Frequency Questionnaire.

**¹** = controlled for sex.

^a^ Sedentary behaviour at weekdays & weekends; ^b^ Physical activity in the previous week.

## Discussion

Our study is unusual in that it has considered two culturally diverse representative samples of middle aged and older Europeans. To allow for the possibility of cultural differences in how demographic factors, lifestyle and psychological well-being interact with obesity, separate models were constructed for Great Britain (GB) and Portugal. The results indicated differences in which factors best explained obesity within the two countries. BMI among the British was explained by having spent less time in education, having reported illness related events, frequent alcohol consumption and lack of physical activity. The finding that BMI in GB was predicted by alcohol consumption and sedentary behaviour is in keeping with accepted science suggesting that obesity is associated with lifestyle [2,8]. The finding that BMI was predicted by having a lower educational level among the GB sample was also as expected and adds to a growing body of evidence suggesting the importance of targeting disadvantaged segments of society in controlling obesity [3,6-8]. The model also suggested that in GB BMI was predicted by having experienced illness themselves or in a close friend or family member, a finding which concurs with existing research which has suggested that informal caring at home can be associated with obesity [38]. The mechanism through which educational disadvantage and/or illness in someone close leads to obesity requires further investigation. Further research is also required to better understand the dynamics linking alcohol intake and obesity in GB. More surprising, the only predictors of BMI among the Portuguese were increasing age and lower resilience. The explanatory power of the BMI models, however, was limited, explaining a mere 6% of the variance in BMI among the British and only 1% among the Portuguese. The weak predictive power for our models of BMI and the disparity between the two countries, as well as with previous studies which have considered psychological factors and BMI, could reflect limitations of BMI as an indicator of obesity [13], particularly in populations of short stature (Table 1) such as the Portuguese.

Waist circumference, on the other hand, has been shown to be closely correlated with visceral fat and other factors associated with the metabolic syndrome [13]. Being male and having spent less time in education predicted greater WC among those in both countries. This apparent sex difference contrasts with previous studies that have identified links between depression and mid-section obesity exclusively in females [16-18,26]. These previous studies and ours, together, highlight the importance of sex in explaining relationships between mid-section obesity and other interacting factors. The finding that having spent fewer years in education predicted a larger WC in both countries agrees with recent research [3,6-8] and implies a need to target the disadvantaged to prevent and treat mid-section obesity. Although education level can be considered a putative marker of deprivation, lack of data on social class that was comparable across the two countries may limit the scope of our models. As expected, given established scientific opinion [1,2], among those in GB WC was associated with sedentary behaviour. Those who reported illness related life events tended to have a larger WC, adding weight to previous observations [38,39] that care providers are more likely to be obese. Consistent with the notion that negative experiences serve to drain resilience [29], illness-related life events and resilience both contributed substantially to WC in the GB sample. It has been argued that awareness of individual vulnerability that may lead to weight gain should be taken into account in health promotion strategies to combat obesity [40]. Assuming that resilience determines how we respond to negative life experiences, the promotion of resilience could reduce such vulnerability. As with BMI, the explanatory power of the GB model (20%) of WC was greater than that of Portugal (6%). The relatively poor explanatory power of the Portuguese model of WC could be a function of the relatively smaller sample size.

Previous research has suggested that certain psychological states can trigger or inhibit eating [41] for example, results from the EPIC study have suggested that depression can be associated with dietary fat consumption [12]. Dietary factors, although associated with obesity, however, did not appear to add to the strength of either the GB or the Portuguese models of BMI or WC. It is difficult to interpret this finding with reference to previous studies of stress [19,20,23,24] or depression [16-19,21,22] and obesity since none appear to have considered dietary intake. It is possible that the finding that dietary habits did not predict obesity in either country could reflect limitations inherent in the self-reported food frequency questionnaire (FFQ). That dietary habits in the current study have been found with the anthropometric measures [42], however, implies that the measure was sensitive enough for the purpose of the study.

Previous studies of obesity and well-being have been biased toward the study of negative psychological states (depression and stress) to the neglect of positive traits and states that may be protective against obesity and which could afford opportunities for intervention. Negative psychological traits did not contribute to either model, bringing into question the large body of research which has indicated a link between WC [17,18,25,26] or BMI [16-18,21] and depression. Existing evidence for a link between depression and obesity is contradictory and our null finding in keeping with several other studies that have found no association between depression and obesity measures [19,22]. Although validated as part of Beck Depression Inventory, that our research considered hopelessness and not depression per se makes it difficult to make direct comparison with previous studies. The current study, in contrast, has explored not only depression and stress, but also the positive traits, mood and resilience. Although a trait inherent in an individual’s personality, resilience can also be construed as a process [32] and as such, has potential to be taught and/or encouraged at the individual level or promoted at group level through the creation of resilience promoting environments. The novel finding that lower resilience predicted higher BMI in Portugal and higher WC in GB suggests that taking measures to promote resilience at both the individual and public health level may reduce obesity in both countries. Resilience could be either or both a driver and a consequence of health behaviour and obesity. That our study has been of cross-sectional design, unfortunately, does not enable us to ascertain the causative nature of co-relationships between variables. Further research is required to better understand the interaction between resilience and other psycho-social and lifestyle factors that may contribute to obesity and impact upon the success of potential intervention.

## Conclusions

Our study is unusual in that it has considered in two culturally diverse representative samples of the middle aged and older normal population, both positive and negative psychological constructs and a range of demographic, social and lifestyle factors that may mediate the relationship between psychological well-being and obesity. That the GB models provided a stronger explanation for WC and BMI compared to Portugal could imply the importance of culture in understanding the interplay between psychosocial, lifestyle and psychological factors in the aetiology of obesity which may go some way toward explaining the mixed findings of previous studies. Both the GB and Portuguese models indicate the importance of targeting males and the less educated and of promoting resilience. Our data suggest that obesity prevention efforts in GB should also target care providers and the unemployed and seek to reduce alcohol consumption and increase physical activity. Meanwhile, our results add weight to the theory that research into positive psychological factors has potential to enhance understanding of obesity.

## Competing interests

The authors declare that they have no competing interests.

## Authors’ contributions

BSK designed the study and drafted the manuscript, MD conducted statistical analyses and contributed to the manuscript, BB directed the statistical analyses, HP conducted preliminary statistical analyses; MDVA co-designed the study and MG led the research project. All authors have read and approved the final manuscript.

## Pre-publication history

The pre-publication history for this paper can be accessed here:

http://www.biomedcentral.com/1471-2458/12/424/prepub
